# Substrate Interference and Strain in the Second-Harmonic Generation from MoSe_2_ Monolayers

**DOI:** 10.1021/acs.nanolett.4c03880

**Published:** 2024-10-02

**Authors:** Sudeep Puri, Sneha Patel, Jose Luis Cabellos, Luis Enrique Rosas-Hernandez, Katlin Reynolds, Hugh O. H. Churchill, Salvador Barraza-Lopez, Bernardo S. Mendoza, Hiroyuki Nakamura

**Affiliations:** † Department of Physics, University of Arkansas, Fayetteville, Arkansas 72701, United States; ‡ Universidad Politécnica de Tapachula, Tapachula, Chiapas C.P. 30830, Mexico; ¶ Centro de Investigaciones en Optica, A.C., León, Guanajuato C.P. 37150, Mexico; § Max Planck Institute for the Structure and Dynamics of Matter, 22761 Hamburg, Germany

**Keywords:** 2D materials, biaxial strain, second-harmonic generation

## Abstract

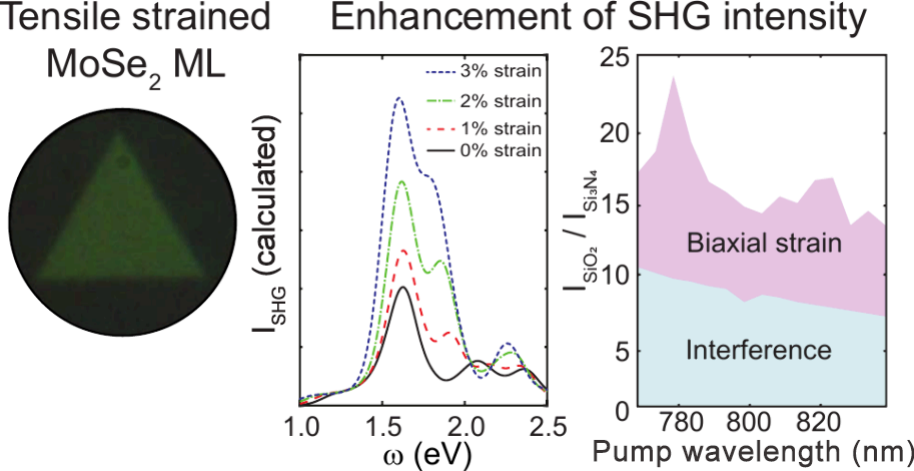

Nonlinear optical materials of atomic thickness, such as non-centrosymmetric 2H transition metal dichalcogenide monolayers, have a second-order nonlinear susceptibility (χ^(2)^) whose intensity can be tuned by strain. However, whether χ^(2)^ is enhanced or reduced by tensile strain is a subject of conflicting reports. Here, we grow high-quality MoSe_2_ monolayers under controlled biaxial strain created by two different substrates and study their linear and nonlinear optical responses with a combination of experimental and theoretical approaches. Up to a 15-fold overall enhancement in second-harmonic generation (SHG) intensity is observed from MoSe_2_ monolayers grown on SiO_2_ when compared to its value on a Si_3_N_4_ substrate. By considering an interference contribution from different dielectrics and their thicknesses, a factor of 2 enhancement of χ^(2)^ was attributed to the biaxial strain: substrate interference and strain are independent handles to engineer the SHG strength of non-centrosymmetric 2D materials.

Second-harmonic generation (SHG) is a nonlinear optical phenomenon in which two photons combine and form a single photon with twice the energy of the original ones.^1^ It is used to determine crystal symmetry^2−6^ and it can also probe excitonic states.^2−6^

Non-centrosymmetric monolayers (MLs) of transition metal dichalcogenides (TMDs) have very high nonlinear susceptibilities of the order of 10^5^–10^8^ pm^2^/V, making them highly efficient for SHG.^2,3,7^ Furthermore, they can sustain large–uniaxial or biaxial–tensile strain.^8,9^ Their crystal symmetry is lowered when under uniaxial strain, resulting in an increased angular anisotropy in polarization-resolved SHG measurements^10−15^ and a decrease in SHG intensity along the armchair direction.^12,15^

As it preserves the point symmetry of the crystal, *biaxial strain* could be a more desirable tool to tune the efficiency of nonlinearity without impacting polarization-dependent properties. Nevertheless, reports on SHG in biaxially tensile strained TMD MLs are scarce and contradictory. For instance, Covre et al. stated a *decrease* in the SHG intensity^16^ on MoSe_2_ MLs, while Liu et al. documented an *enhancement* in SHG in a MoS_2_ ML.^17^ Similarly, Shi et al. observed an increased SHG in atomically thin WS_2_ produced on silicon holes, which was explained as a result of a Fabry–Perot cavity resonance.^18^ Adding to the conflicting nature of some reports, the tuning rate for uniaxial strain in ref^12^ was used to rule out the involvement of strain, even though the experimental geometry appears to more closely match one in which the 2D material is under biaxial strain.^18^

To resolve this puzzle, biaxially strained MoSe_2_ MLs were grown by physical vapor deposition on Si_3_N_4_ and SiO_2_ substrates here. Strain was induced by a rapid thermal quenching after growth. The biaxial nature of strain as well as its magnitude were determined by optical characterization that included photoluminescence (PL), Raman studies, and theory. Details follow.

A high growth temperature in the PVD process efficiently induces a tensile strain due to the thermal expansion mismatch between the ML and the substrate. We used two types of substrates: Si_3_N_4_ and SiO_2_ dielectrics grown on Si wafers; see schematics in Figure 1(a). The percent of strain induced due to the thermal expansion mismatch is given by^19^
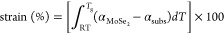
1where α_MoSe_2__ = 7.2 × 10^–6^ is the thermal expansion coefficient of bulk MoSe_2_^20^ which we assumed to be a temperature independent constant. α_subs_ can either be ,^21^ or α_SiO_2__ = 5.0 × 10^–7^.^22^*T*_g_ is the growth temperature and RT stands for room temperature. Additional details will be presented elsewhere.^37^ Our estimates yield up to 0.43%, or 0.73% tensile strain at *T*_g_ = 1130 °C when the substrate is Si_3_N_4_ or SiO_2_, respectively. (See Methods for details.)

**Figure 1:**
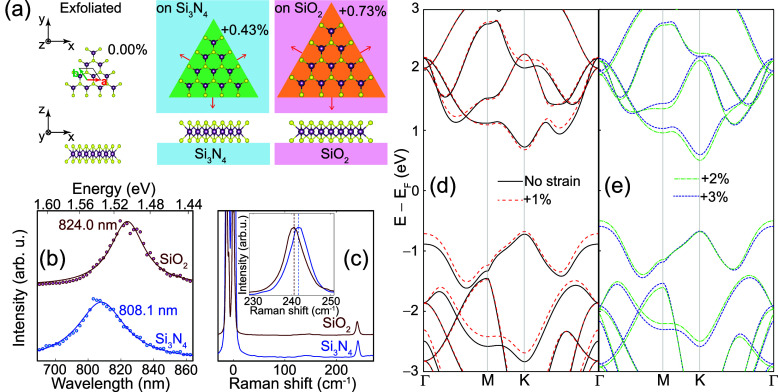
(a) Schematics of MoSe_2_ MLs without strain and under biaxial tensile strain originating by a rapidly quenched growth on Si_3_N_4_ or SiO_2_ substrates. The disposition of the unit cell employed in SHG calculations and Cartesian axes are depicted in the leftmost diagrams. (b) PL of MoSe_2_ MLs grown on Si_3_N_4_ and SiO_2_. Experimental data (dots) with Lorentzian fits (solid lines) show red-shift of the exciton peak on SiO_2_ from biaxial tensile strain. (c) Raman shift in MoSe_2_ MLs on Si_3_N_4_ and SiO_2_ substrates. The inset highlights the *A*_1*g*_ phonon peak for the MLs on each substrate, which is known to red-shift for biaxial strain^23^ but not for uniaxial strain. (d) PBE-DFT band structures of a MoSe_2_ ML under no strain (solid lines) or 1% tensile strain; the direct nature of the band gap (at the *K*-point) is apparent. (e) Electronic band structure under a 2% and 3% tensile strain.

When compared to unstrained MLs typically created by exfoliation, the PL shows a red-shift of the exciton peak for samples grown on Si_3_N_4_ and SiO_2_ substrates [Figure 1(b)], which is a signature of biaxial tensile strain. In turn, the Raman shift measured for MoSe_2_ MLs on both substrates also shows a red-shifted *A*_1*g*_ phonon peak (known to be significant only under biaxial strain^23^) in Figure 1(c). To further assess the existence of strain, MoSe_2_ MLs grown on SiO_2_/Si were transferred to a fresh SiO_2_/Si substrate of the same thickness to release the strain present on the sample (see Figure S1 and Section 1 of the Supporting Information). As-grown (strained) MoSe_2_ MLs showed a significant red-shift in both PL and *A*_1*g*_ Raman mode compared to the transferred (unstrained) MLs (see Figures S2(a) and S2(b) in Section 2 of the Supporting Information), reaffirming the presence of biaxial strain in the as-grown MoSe_2_. Furthermore, PL and Raman spectra were acquired on strained samples grown at 1030 and 1075 °C (Section 3 in the Supporting Information). As depicted in Figure 1(d), MoSe_2_ MLs display a direct bandgap at the *K*-point when under no strain, which reduces in size under a 1% biaxial tensile strain. As seen in Figure 1(e), further (2 and 3%) tensile strain induces an indirect bandgap (in between the Γ and *K* points)^24^ (seeMethods).

We now turn to a SHG measured by a setup schematically shown in Figure 2(a). The excitation source is a Ti:sapphire femtosecond laser (Tsunami, Spectra-Physics) generating 200 fs pulses at a repetition rate of 80 MHz (see Methods). An inset in Figure 2(a) shows a log–log plot of pump power versus SHG intensity of the MoSe_2_ ML with a slope of 2, which is a hallmark of SHG. For polarization-resolved SHG measurements, the sample is rotated in 5° increments, and a linear polarizer is used in the detection line to collect SHG signals with a polarization parallel to that of the pump laser. The polarization resolved SHG depicted in Figure 2(b) shows a characteristic 6-fold symmetry that arises from the *D*_3*h*_ symmetry of the underlying crystal,^2,11,25^*even under biaxial strain*.

**Figure 2:**
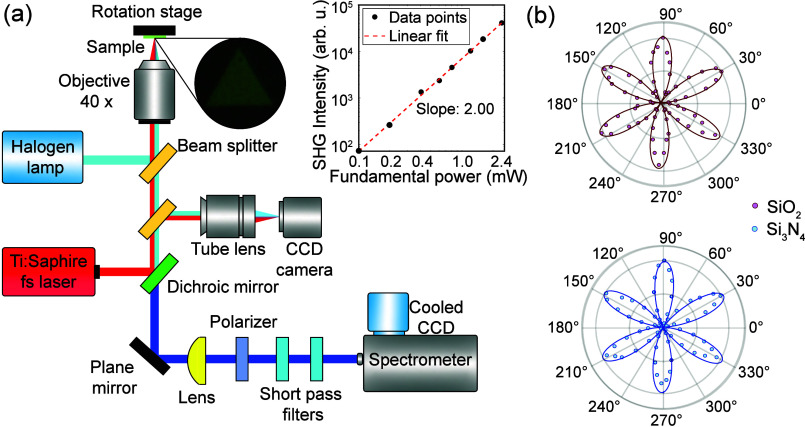
(a) Sketch of the experimental setup for the collection of SHG light in reflection geometry using a tunable femtosecond laser. The inset shown is a double logarithmic plot of pump power dependence of SHG. The slope of the plot is 2. (b) *Normalized* SHG intensity as a function of sample rotation angle θ about the *z*-axis, in which the polarization of the SHG signal is parallel to that of the pump laser. Experimental data points are represented by pink dots for MoSe_2_ MLs on SiO_2_ and by blue dots for MoSe_2_ MLs on Si_3_N_4_, and the solid line is proportional to sin^2^(3θ).

Following Wang and Qian,^26^ we calculate the effective second order susceptibility for a parallel polarization configuration by expanding the expression for the SHG polarization,
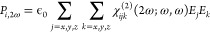
2where ϵ_0_ is the permittivity. There is only one independent entry in the second-order dielectric susceptibility for point group 6̅m2 (*D*_3*h*_):^1^ χ_*yyy*_^(2)^ = −χ_*yxx*_^(2)^ = −χ_*xxy*_^(2)^ = −χ_*xyx*_^(2)^ ≡ χ^(2)^, where the *x*, *y* and *z* labels correspond to the laboratory axes as shown in Figure 1(a) and we dropped the dependency on 2ω and ω from χ^(2)^. The second-order (harmonic) polarization takes the form:
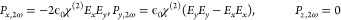
3In a polarization-resolved SHG, an analyzer is inserted to select SHG polarized either parallel or perpendicular to that of the pump beam, while rotating the polarization of the pump on the sample. The electric field of the pump at an angle of θ with respect to the zigzag (*x*) direction of MoSe_2_ ML can be expressed as **E** = (*E*_0_ cos θ, *E*_0_ sin θ, 0). The effective second order susceptibility in the parallel configuration χ_∥_^(2)^ (see ref (26)) is obtained by applying a projection onto a vector parallel to the polarization of the incoming beam:
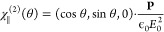
4with **P** as in Equation 3. This way,
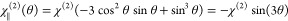
5and the SHG intensity in the polar plot [solid curves in Figure 2(b)] can be expressed as
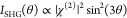
6The SHG intensity is thus proportional to |χ^(2)^|^2^.

At this point, we address the main contribution from this work, i.e., the *relative magnitude* of the SHG signals obtained on the two biaxially strained samples. To this end, Figures 3(a) and 3(b) show the SHG intensity as a function of the SHG wavelength for the tensile strained ML samples grown on Si_3_N_4_ and SiO_2_ substrates, respectively. The overall increase in the SHG intensity at longer wavelengths resembles earlier results.^27,28^ We also observed a shoulder on the SHG spectrum, which is attributed to single photon resonances known as A-excitons at pump wavelengths of 805 and 810 nm for MoSe_2_ MLs on Si_3_N_4_ and SiO_2_, respectively. The different shifts in A-exciton peaks confirm the different biaxial tensile strain on the two different substrates discussed in Figures 1(b) and 1(c). The rise of SHG intensity for wavelengths over 416–420 nm is consistent with the resonant features (C-exciton peaks) expected from interband transitions in MoSe_2_ MLs.^15,27^

**Figure 3:**
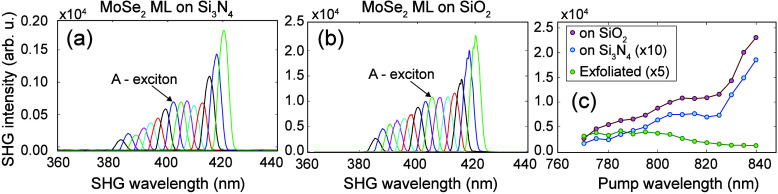
(a) SHG intensity as a function of the SHG wavelength for the ML sample on Si_3_N_4_. (b) SHG intensity as a function of the SHG wavelength for the ML sample and SiO_2_. The shoulders on the SHG spectrum in (a) and (b) are attributed to single-photon resonance at the A-exciton at around the pump wavelengths of 805 and 810 nm for MoSe_2_ MLs on Si_3_N_4_ and SiO_2_, respectively, which is consistent with a different biaxial tensile strain being created by the two different substrates. (c) A 15-fold enhancement in the SHG response from the MoSe_2_ ML on SiO_2_ when contrasted to that on Si_3_N_4_ across the measured wavelengths.

A ratio of SHG intensity across the measured wavelength range is provided in Figure 3(c). The main takeaway is a large, *nearly 15-fold enhancement in the SHG response* of the MoSe_2_ ML on 87 nm thick SiO_2_ when compared to that of a MoSe_2_ ML on 73 nm thick Si_3_N_4_. On the other hand, quantitatively comparing the SHG intensity of strained MoSe_2_ MLs with the exfoliated ML poses a challenge because (1) intrinsic material properties (such as doping) may differ between the exfoliated versus PVD-grown MoSe_2_ MLs, and (2) the 285 nm thick SiO_2_ substrate used for the exfoliated ML happens to be close to a minimum of interference, which makes the interference contribution highly sensitive to a small error in the real thickness of the dielectric layer. Instead, a comparison of SHG intensity between the as-grown (strained) MLs with the transferred (unstrained) ones is provided in Figure S2(c), in which the as-grown ML yielded larger SHG intensity than the transferred ones by approximately a factor of 2. The value of χ^(2)^ for a strained MoSe_2_ ML was estimated by comparing with a quartz reference to be 1.7 × 10^6^ pm^2^/V or 2,700 pm/V; details can be found in Section 4 of the Supporting Information. In what follows, we focus on PVD grown MoSe_2_ ML samples to determine the contribution of strain on the observed SHG enhancement, combining experimental data and computationally obtained SHG responses under strain.

To calculate the effect of substrate interference on the SHG response, we use the model by Song et al.^29^ For the normally incident pump of wavelength λ and intensity *I*_λ_ on MoSe_2_ ML on a dielectric film of thickness *d* and a Si substrate, the SHG intensity *I*_λ/2_ is given by^29^
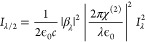
7where β_λ_ is the structure factor that encapsulates the influences arising from the layered structure and is given as
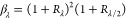
8Here *R*_λ_ and *R*_λ/2_ are the reflection coefficients of whole structure of λ and λ/2 which are given by
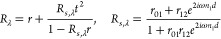
9

In addition, *r* = −η/(1 + η) and *t* = 1/(1 + η) are the reflection and transmission coefficients of a MoSe_2_ ML, respectively, and η is calculated from the effective bulk refractive index *n*_2*D*_ as η=-*ihω*(*n*_2*D*_^2^-1)/2 with ω = 2π/λ. The reflection coefficient at the interface between two layers is given by *r*_*ij*_ = (*n*_*i*_ – *n*_*j*_)/(*n*_*i*_ + *n*_*j*_).

The enhancement factor due to interference (EF_int_) is thus written as
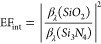
10and displayed in Figure 4(a) for the two substrates used here. The calculation assumes MoSe_2_ ML on SiO_2_(*d* nm)/Si or Si_3_N_4_(*d* nm)/Si substrates as a function of *d* under an 800 nm pump laser. The delineated gray dashed lines serve as indicators for the enhancement resulting from interference, corresponding to 50, 87, 200, and 300 nm (73 nm) thick SiO_2_ (Si_3_N_4_) substrates utilized in our experiment (see Section 5 in the Supporting Information). The results indicate that an EF_int_ up to 7.2 (for 87 nm-thick SiO_2_) could be attributable to constructive interference (see Section 6 in the Supporting Information). We note that the interference could be overestimated by about 20–30% due to the use of plane waves in the calculation, as the experiment focuses beam through the high numerical aperture (NA) objective lens.^29^

**Figure 4:**
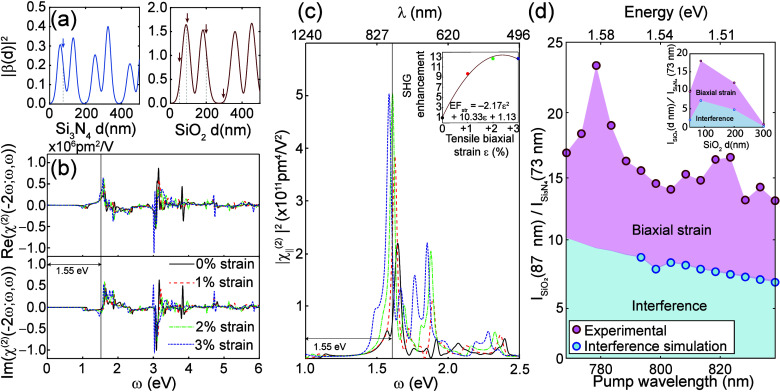
(a) Substrate interference β^2^ versus dielectric thickness *d* for Si_3_N_4_ (left subplot) and SiO_2_ (right subplot) slabs. The gray lines highlight the thickness of the dielectric films used in our experiments. (b) Real and imaginary contributions to χ^(2)^(ω). (c) Evolution of the SHG intensity due to biaxial strain alone, as a function of frequency. (EF_str_ in the inset is the enhancement factor due to biaxial tensile strain for ω = 1.55 eV.) (d) Experimental observation of SHG relative enhancement and elucidation of the separate effects of interference and biaxial strain. The inset shows the SHG enhancement factor on SiO_2_ substrates of different thicknesses evaluated at an 800 nm (1.55 eV) pump wavelength.

The real and imaginary parts of χ^(2)^ as a function of ω calculated with the TINIBA code (developed by J. L. Cabellos, T. Rangel and B. Mendoza) are provided in Figure 4(b). In Figure 4(c), we depict the energy dependency of |χ^(2)^|^2^ (proportional to *I*_SHG_), which is seen to increase monotonically with applied tensile biaxial strain at excitation energies (1.48–1.61 eV) studied by the experiment (Figure 4(d)). Excitingly, *I*_*SHG*_ is of the same order as the experimental estimate in Section 4 of the Supporting Information.

Theory reproduces the tensile-strain induced *enhancement* of SHG by the experiment up to every detail. Furthermore, both theory and experiment highlight the importance of resonant features that originate from band-to-band transitions, as seen by steeply changing SHG intensity as a function of wavelength. The theory also suggests that even qualitatively different strain effects may be expected in other wavelength ranges near the resonance, which may explain widely varying strain effects in the literature.

In summary, we explain two independent contributors to the enhancement of SHG in non-centrosymmetric crystals: biaxial tensile strain, and interference originating at the supporting substrate. This way, we provide clarity on two independent factors affecting the magnitude of the SHG on 2D non-centrosymmetric crystals unequivocally. These results shed light on the biaxial strain tuning of nonlinear optical process in atomically thin materials, and provide invaluable guidelines for future nanoscale nonlinear/quantum optical devices.^35,36^

## Methods

### Experimental Section

MoSe_2_ MLs were grown by physical vapor deposition (PVD) at 1030–1130 °C. We used MoSe_2_ from AlfaAser (99.9% pure) as a precursor under argon flow. The diameter of a quartz tube was 1 in.. We employed reverse flow of argon from substrate toward the powder end to avoid any unwanted nucleation before the growth temperature is reached. Once the growth temperature is reached, forward flow is used for a short time (typically 10 s) for growth and then switched back to the reverse flow. Rapid quenching of the growth was carried out by sliding the quartz tube out of the hot zone of the furnace. As a comparison, we also measured exfoliated MoSe_2_ MLs purchased from a vendor (2D semiconductors) on SiO_2_. Additional details on sample preparation including optical contrast on different SiO_2_ substrates and transfer of as-grown samples to release strain are shown in Sections 1 and 5 of the Supporting Information.

For the polarization resolved SHG setup, we used a Ti:sapphire femtosecond (fs) laser source (Tsunami, Spectra-Physics) generating pulses of duration 200 fs at repetition rate of 80 MHz. With the use of a prism compensator, the pulse width was kept constant between 770 and 840 nm. A dichroic mirror directs excitation laser to 40x objective lens (NA 0.6) to excite the sample and the SHG signal is collected in the reflection geometry. A beam splitter added on the beam path illuminates the sample with white light and another beam splitter directs the reflected light to a CCD camera for sample imaging. Two short pass filters were used to reject the pump beam to detect SHG signals in a 500 mm focal spectrometer (Acton Research) equipped with a thermoelectrically cooled CCD. For the polarization resolved SHG measurements, sample is rotated in 5° steps and a linear polarizer is used in the detection line to collect SHG signals parallel to the pump polarization. When comparing the relative intensity of SHG, any data involved was taken consecutively on the same day using identical measurement conditions. This approach eliminates the impact of any unintentional difference in measurement conditions that may originate from a small change in optical alignment or laser pulse conditions.

### Theoretical

First-principles calculations were carried out using plane wave density functional theory (DFT) as implemented in the ABINIT^30^ package. The General Gradient Approximation (GGA) with Perdew–Burke–Ernzerhof^31^ (PBE) functional was considered, along with optimized norm-conserving Vanderbilt pseudopotentials.^32^ The plane-wave energy cutoff was set to 20 hartree (500 eV) and a Monkhorst–Pack^33^*k*-point sampling of 18 × 18 × 1 centered in the Γ point was used. The results indicate that the pristine MoSe_2_ ML has a lattice constant of *a* = 3.327 Å, a distance between selenium bonds of *d*_*Se*–*Se*_ = 3.344 Å, and a band gap of *E*_*g*_ = 1.445 eV, which are in good agreement with previously reported values.^23,34^ After considering a biaxial tensile strain of 1%, 2% and 3% we found that at 1% the band gap remains direct as in the pristine case but undergoes from direct to indirect as more strain is applied as predicted before.^23^ The second order susceptibility tensor was calculated with the TINIBA code, developed at the Centro de Investigaciones en Optica by B. Mendoza, J. L. Cabellos, and T. Rangel.

We used a scissor correction equal to 0.809 eV on the electronic band structure of all calculated susceptibilities; this value was taken as the GW gap reported in ref (24). A Gaussian broadening of 0.025 eV (300 K) was used in the calculations. Our calculations are of the single-particle type, so the calculated responses do not include excitonic effects.

## Supporting Information Available

Additional details on the ML transfer process, optical characterizations, experimental estimation of χ^(2)^, and the SiO_2_ thickness dependence of SHG (PDF)

## References

[ref1] Boyd R. W. Nonlinear Optics, 4th ed.; Academic Press, 2020.

[ref2] Malard L. M.; Alencar T. V.; Barboza A. P. M.; Mak K. F.; De Paula A. M. Observation of intense second harmonic generation from MoS_2_ atomic crystals. Phys. Rev. B 2013, 87, 201401 10.1103/PhysRevB.87.201401.

[ref3] Kumar N.; Najmaei S.; Cui Q.; Ceballos F.; Ajayan P. M.; Lou J.; Zhao H. Second harmonic microscopy of monolayer MoS_2_. Phys. Rev. B 2013, 87, 161403 10.1103/PhysRevB.87.161403.

[ref4] Shi J.; Yu P.; Liu F.; He P.; Wang R.; Qin L.; Zhou J.; Li X.; Zhou J.; Sui X.; et al. 3R MoS2 with broken inversion symmetry: a promising ultrathin nonlinear optical device. Adv. Mater. 2017, 29, 1701486 10.1002/adma.201701486. 28590583

[ref5] Klimmer S.; Ghaebi O.; Gan Z.; George A.; Turchanin A.; Cerullo G.; Soavi G. All-optical polarization and amplitude modulation of second-harmonic generation in atomically thin semiconductors. Nat. Photonics 2021, 15, 837–842. 10.1038/s41566-021-00859-y.

[ref6] Hsu W.-T.; Zhao Z.-A.; Li L.-J.; Chen C.-H.; Chiu M.-H.; Chang P.-S.; Chou Y.-C.; Chang W.-H. Second Harmonic Generation from Artificially Stacked Transition Metal Dichalcogenide Twisted Bilayers. ACS Nano 2014, 8, 2951–2958. 10.1021/nn500228r. 24568359

[ref7] Wen X.; Gong Z.; Li D. Nonlinear optics of two-dimensional transition metal dichalcogenides. InfoMat 2019, 1, 317–337. 10.1002/inf2.12024.

[ref8] Roldán R.; Castellanos-Gomez A.; Cappelluti E.; Guinea F. Strain engineering in semiconducting two-dimensional crystals. J. Phys.: Condens. Matter 2015, 27, 313201 10.1088/0953-8984/27/31/313201. 26199038

[ref9] Naumis G. G.; Herrera S. A.; Poudel S. P.; Nakamura H.; Barraza-Lopez S. Mechanical, electronic, optical, piezoelectric and ferroic properties of strained graphene and other strained monolayers and multilayers: an update. Rep. Prog. Phys. 2024, 87, 016502 10.1088/1361-6633/ad06db. 37879327

[ref10] Mennel L.; Paur M.; Mueller T. Second harmonic generation in strained transition metal dichalcogenide monolayers: MoS_2_, MoSe_2_, WS_2_, and WSe_2_. APL Photonics 2019, 4, 10.1063/1.5051965.

[ref11] Mennel L.; Furchi M. M.; Wachter S.; Paur M.; Polyushkin D. K.; Mueller T. Optical imaging of strain in two-dimensional crystals. Nat. Commun. 2018, 9, 516 10.1038/s41467-018-02830-y. 29410470 PMC5802795

[ref12] Liang J.; Zhang J.; Li Z.; Hong H.; Wang J.; Zhang Z.; Zhou X.; Qiao R.; Xu J.; Gao P.; et al. Monitoring local strain vector in atomic-layered MoSe_2_ by second-harmonic generation. Nano Lett. 2017, 17, 7539–7543. 10.1021/acs.nanolett.7b03476. 29164881

[ref13] Li Z.-Y.; Cheng H.-Y.; Kung S.-H.; Yao H.-C.; Inbaraj C. R. P.; Sankar R.; Ou M.-N.; Chen Y.-F.; Lee C.-C.; Lin K.-H. Uniaxial Strain Dependence on Angle-Resolved Optical Second Harmonic Generation from a Few Layers of Indium Selenide. Nanomaterials 2023, 13, 750 10.3390/nano13040750. 36839118 PMC9962579

[ref14] Khan A. R.; Liu B.; Lu T.; Zhang L.; Sharma A.; Zhu Y.; Ma W.; Lu Y. Direct measurement of folding angle and strain vector in atomically thin WS_2_ using second-harmonic generation. ACS Nano 2020, 14, 15806–15815. 10.1021/acsnano.0c06901. 33179915

[ref15] Mennel L.; Smejkal V.; Linhart L.; Burgdórfer J.; Libisch F.; Mueller T. Band nesting in two-dimensional crystals: An exceptionally sensitive probe of strain. Nano Lett. 2020, 20, 4242–4248. 10.1021/acs.nanolett.0c00694. 32436711 PMC7291349

[ref16] Covre F. S.; Faria P.; Gordo V. O.; de Brito C. S.; Zhumagulov Y. V.; Teodoro M. D.; Couto O.; Misoguti L.; Pratavieira S.; Andrade M. B. d.; et al. Revealing the impact of strain in the optical properties of bubbles in monolayer MoSe_2_. Nanoscale 2022, 14, 5758–5768. 10.1039/D2NR00315E. 35348558

[ref17] Liu B.; Yildirim T.; Blundo E.; de Ceglia D.; Khan A. R.; Yin Z.; Nguyen H. T.; Pettinari G.; Felici M.; Polimeni A.; Extraordinary second harmonic generation modulated by divergent strain field in pressurized monolayer domes. Appl. Phys. Rev. 2023, 10, 10.1063/5.0144641.

[ref18] Shi J.; Wu X.; Wu K.; Zhang S.; Sui X.; Du W.; Yue S.; Liang Y.; Jiang C.; Wang Z.; et al. Giant Enhancement and Directional Second Harmonic Emission from Monolayer WS_2_ on Silicon Substrate via Fabry-Perot Micro-Cavity. ACS Nano 2022, 16, 13933–13941. 10.1021/acsnano.2c03033. 35984986

[ref19] Ahn G. H.; Amani M.; Rasool H.; Lien D.-H.; Mastandrea J. P.; Ager Iii J. W.; Dubey M.; Chrzan D. C.; Minor A. M.; Javey A. Strain-engineered growth of two-dimensional materials. Nat. Commun. 2017, 8, 608 10.1038/s41467-017-00516-5. 28931806 PMC5606995

[ref20] El-Mahalaey S. H.; Evans B. The Thermal Expansion of 2H-MoS_2_, *2H*-MoSe_2_ and *2H*-WSe_2_ between 20 and 800 °C. J. Appl. Crystallogr. 1976, 9, 403–406. 10.1107/S0021889876011709.

[ref21] Sinha A. K.; Levinstein H. J.; Smith T. E. Thermal stresses and cracking resistance of dielectric films (SiN, Si_3_N_4_, and SiO_2_) on Si substrates. J. Appl. Phys. 1978, 49, 2423–2426. 10.1063/1.325084.

[ref22] Rustum Roy D. K. A.; McKinstry H. A. Very Low Thermal Expansion Coefficient Materials. Annu. Rev. Matter. Sci. 1989, 19, 59–81. 10.1146/annurev.ms.19.080189.000423.

[ref37] Patel S.; Faltermeier T.; Puri S.; Rodriguez R.; Reynolds K.; Davari S.; Churchill H. O. H.; Borys N. J.; Nakamura H. Biaxial strain tuning of excitons in monolayer MoSe_2_ by high-temperature physical vapor deposition. arXiv 2024, arXiv:2408.15469, 10.48550/arXiv.2408.15469.

[ref23] Horzum S.; Sahin H.; Cahangirov S.; Cudazzo P.; Rubio A.; Serin T.; Peeters F. M. Phonon softening and direct to indirect band gap crossover in strained single-layer MoSe_2_. Phys. Rev. B 2013, 87, 125415 10.1103/PhysRevB.87.125415.

[ref24] Ugeda M. M.; Bradley A. J.; Shi S.-F.; da Jornada F. H.; Zhang Y.; Qiu D. Y.; Ruan W.; Mo S.-K.; Hussain Z.; Shen Z.-X.; et al. Giant bandgap renormalization and excitonic effects in a monolayer transition metal dichalcogenide semiconductor. Nat. Mater. 2014, 13, 1091–1095. 10.1038/nmat4061. 25173579

[ref25] Kim D. H.; Lim D. Optical second-harmonic generation in few-layer MoSe_2_. JKPS 2015, 66, 816–820. 10.3938/jkps.66.816.

[ref26] Wang H.; Qian X. Giant optical second harmonic generation in two-dimensional multiferroics. Nano Lett. 2017, 17, 5027–5034. 10.1021/acs.nanolett.7b02268. 28671472

[ref27] Kikuchi Y.; Miyauchi Y.; Takaoka R.; Suzuki T.; Tanaka M.; Ohno S. Multiple-peak resonance of optical second harmonic generation arising from band nesting in monolayer transition metal dichalcogenides TX_2_ on SiO_2_/Si (001) substrates (T= Mo, W; X= S, Se). Phys. Rev. B 2019, 100, 075301 10.1103/PhysRevB.100.075301.

[ref28] Miyauchi Y.; Morishita R.; Tanaka M.; Ohno S.; Mizutani G.; Suzuki T. Influence of the oxide thickness of a SiO_2_/Si (001) substrate on the optical second harmonic intensity of few-layer MoSe_2_. JJAP 2016, 55, 085801 10.7567/JJAP.55.085801.

[ref29] Song Y.; Wang W.; Wang Y.; Shan Y.; Cheng J. L.; Sipe J. Interference tunable second harmonic generation for two-dimensional materials in layered structures. Opt. Expr. 2023, 31, 19746–19753. 10.1364/OE.486719. 37381383

[ref30] Gonze X.; Amadon B.; Anglade P. M.; Beuken J. M.; Bottin F.; Boulanger P.; Bruneval F.; Caliste D.; Caracas R.; Cote M.; et al. ABINIT: First-principles approach to material and nanosystem properties. Comput. Phys. Commun. 2009, 180, 2582–2615. 10.1016/j.cpc.2009.07.007.

[ref31] Perdew J. P.; Burke K.; Ernzerhof M. Generalized Gradient Approximation Made Simple. Phys. Rev. Lett. 1996, 77, 3865–3868. 10.1103/PhysRevLett.77.3865. 10062328

[ref32] Hamann D. R. Optimized norm-conserving Vanderbilt pseudopotentials. Phys. Rev. B 2013, 88, 085117 10.1103/PhysRevB.88.085117.

[ref33] Monkhorst H. J.; Pack J. D. Special points for Brillouin-zone integrations. Phys. Rev. B 1976, 13, 5188–5192. 10.1103/PhysRevB.13.5188.

[ref34] Chang C.-H.; Fan X.; Lin S.-H.; Kuo J.-L. Orbital analysis of electronic structure and phonon dispersion in MoS_2_, MoSe_2_, WS_2_, and WSe_2_ monolayers under strain. Phys. Rev. B 2013, 88, 195420 10.1103/PhysRevB.88.195420.

[ref35] Moody G.; Sorger V. J.; Blumenthal D. J.; Juodawlkis P. W.; Loh W.; Sorace-Agaskar C.; Jones A. E.; Balram K. C.; Matthews J. C. F.; Laing A.; et al. 2022 Roadmap on integrated quantum photonics. J. Phys: Photon. 2022, 4, 012501 10.1088/2515-7647/ac1ef4.

[ref36] Autere A.; Jussila H.; Dai Y.; Wang Y.; Lipsanen H.; Sun Z. Nonlinear optics with 2D layered materials. Adv. Mater. 2018, 30, 1705963 10.1002/adma.201705963. 29575171

