# Supplementary material for: Substrate Interference and Strain in the Second-Harmonic Generation from MoSe2 Monolayers
Source: Nano Lett. 2024 Oct 2;24(41):13061–7. doi: 10.1021/acs.nanolett.4c03880 (PMC11487631; doi:10.1021/acs.nanolett.4c03880)
Supplement: Supplementary file 1 — nl4c03880_si_001.pdf [file nl4c03880_si_001.pdf]

Supporting information for:

Substrate interference and strain in the second harmonic generation from $MoSe_2$ monolayers

Sudeep Puri[1], Sneha Patel[1], Jose Luis Cabellos[2], Luis Enrique Rosas-Hernandez[1], Katlin Reynolds[1], Hugh O. H. Churchill[1], Salvador Barraza-Lopez[1], Bernardo S. Mendoza[3,4], and Hiroyuki Nakamura[1*]

[1]*Department of Physics, University of Arkansas, Fayetteville, AR 72701, USA*

[2]*Universidad Politécnica de Tapachula. C.P. 30830. Tapachula, Chiapas, Mexico*

[3]*Centro de Investigaciones en Optica, A.C., León, C.P. 37150. Guanajuato, Mexico*

[4]*Max Planck Institute for the Structure and Dynamics of Matter, 22761 Hamburg, Germany*

Corresponding author: hnakamur@uark.edu

Table of Contents

Section	Page
1. Transfer of PVD-grown $MoSe_2$ monolayer islands.	2
2. Optical characterization of as-grown and transferred $MoSe_2$ monolayers.	3
3. Optical characterization of monolayer $MoSe_2$ monolayers grown at two temperatures.	3
4. Experimental estimation of $\chi^{(2)}$ for $MoSe_2$ monolayers.	4
5. Optical images of $MoSe_2$ monolayers on SiO_2/Si with different thicknesses.	5
6. Thickness dependence (SiO_2 substrate) on the second harmonic generation.	6

1. Transfer of PVD-grown $MoSe_2$ monolayer islands

We used the nail polish transfer technique [1] to transfer PVD-grown $MoSe_2$ monolayer islands grown on a 87 nm SiO_2/Si substrate onto a fresh 87 nm SiO_2/Si substrate. The strain is released after transfer. First, a small square-cut polydimethylsiloxane (PDMS) was placed on the edge of a glass slide which is then covered with scotch tape. A drop of nail polish is placed on the PDMS, and the slide is annealed at 90°C for 5 min afterwards. The nail polish stamp is then pressed over the flake to be transferred from the growth substrate, and the substrate is heated to 80°C. The system is then allowed to cool down to room temperature and the nail polish stamp is raised to pick up the flake from the growth substrate. The nail polish stamp is then pressed over a fresh Si substrate with 87 nm of thermally grown SiO_2. The substrate is heated to 120°C, and the nail polish stamp is pulled up slowly afterwards, leaving behind $MoSe_2$ monolayer islands and nail polish on

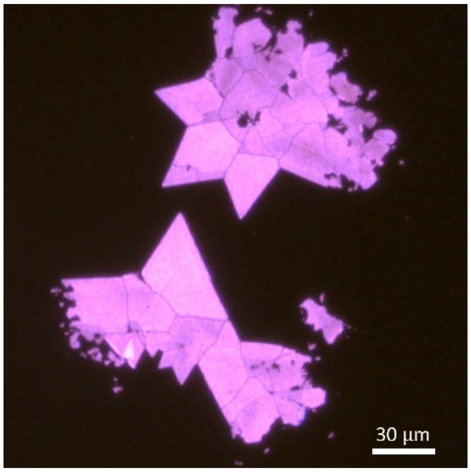

Figure S1. PL image of transferred PVD-grown $MoSe_2$ monolayer flakes.

the substrate. The nail polish is removed using acetone and isopropyl alcohol. Figure S1 shows the PL image of the transferred island.

2. Optical characterization of as-grown and transferred $MoSe_2$ monolayers

PL, Raman, and SHG measurements were carried out on (i) as-grown (strained) $MoSe_2$ monolayers and (ii) after transferring MoS_2 from the same growth batch onto another fresh SiO_2/Si substrate to create unstrained monolayers. Both the as-grown and transferred $MoSe_2$ monolayers were on a 87 nm SiO_2/Si substrate. We observed a significant red shift, about 30 nm (57 meV) in the PL peak of as-grown $MoSe_2$ monolayers when compared to the transferred ones; see Figure S2(a). Similarly, we observed a softening of A_{1g} Raman mode by ~2.12 cm^{-1} in an as-grown ML compared to a transferred one (Figure S2(b)). The PL and Raman peaks of the transferred (unstrained) $MoSe_2$ monolayers are at about 1.57 eV and 240.09 cm^{-1}, respectively, which is in good agreement with the unstrained values from literature: 1.59 eV [2] and 240.5 cm^{-1} [3], respectively. This confirms the presence of strain in as-grown $MoSe_2$ and the release of strain in the transferred one. The as-grown (strained) $MoSe_2$ on SiO_2 showed SHG higher than the transferred one by approximately a factor of two [Figure S2(c)], substantiating the enhancement of second order non-linearity in biaxially tensile strained MLs. The two SHG measurements were performed back-to-back on the same day, using the same optical alignment and laser pulse conditions, to eliminate the impact of any unintentional difference in measurement conditions.

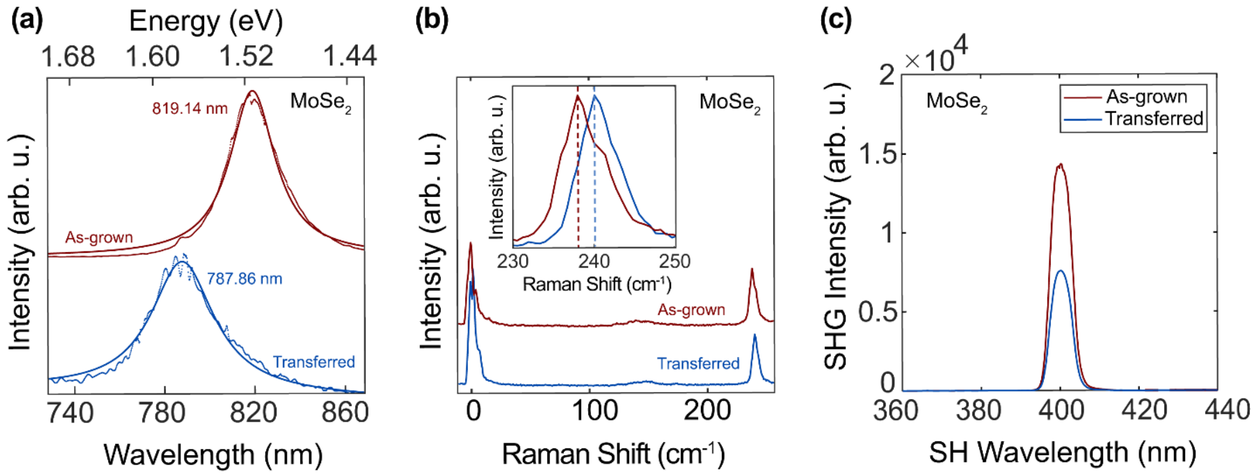

Figure S2. Optical characterization of as-grown and transferred $MoSe_2$ monolayers. Comparative (a) PL (b) Raman, and (c) SHG plot of as-grown (strained) and transferred (unstrained) $MoSe_2$ monolayers.

3. Optical characterization of $MoSe_2$ monolayers grown at two temperatures

Additional $MoSe_2$ monolayers were grown on an 87 nm SiO_2/Si substrate at two different temperatures: 1030 °C, and 1075 °C. We observed a small redshift of about 4 nm (25 meV) on $MoSe_2$ monolayers grown at 1075 °C when compared to the ones grown at 1030 °C. Additionally, we observed a slight softening (0.3 cm^{-1}) of the A_{1g} Raman mode for $MoSe_2$ monolayers grown at

1030 °C when compared to those grown at 1075 °C. Furthermore, we observed a ~30% increase in the SHG intensity for $MoSe_2$ monolayers grown at 1075 °C when compared to those grown at 1030 °C. The very small differences between the two monolayers likely reflect the relatively small change in the expected biaxial strain: +0.70% (1075 °C) vs. +0.67% (1030 °C). See Figure S3.

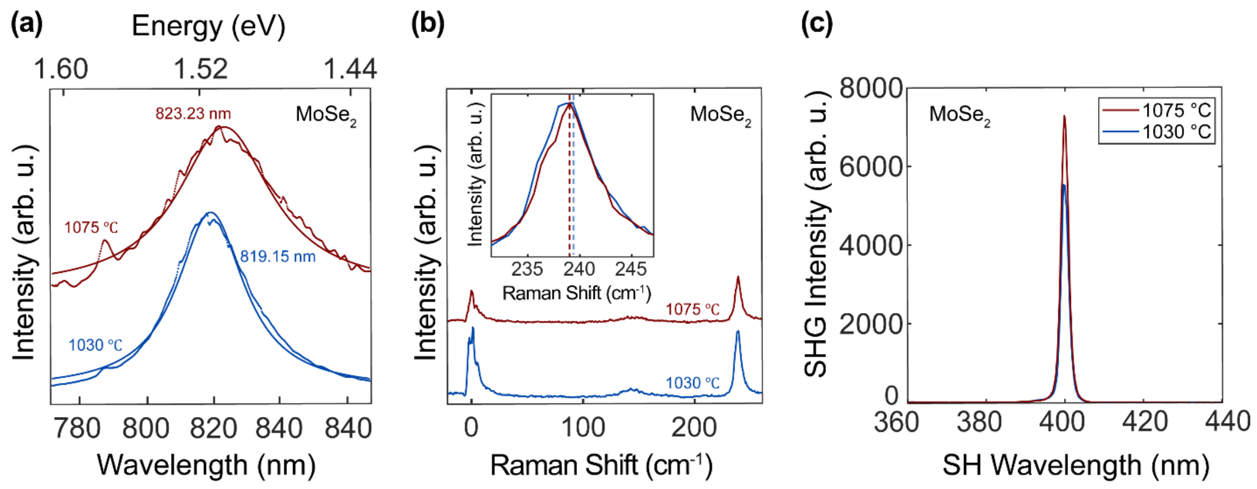

Figure S3. Optical characterization of monolayer $MoSe_2$ grown at 1030°C, and 1075°C. Comparative (a) PL plots (b) Raman plots, and (c) SHG plots of $MoSe_2$ monolayers grown at 1030 °C and 1075 °C.

4. Experimental estimation of $\chi^{(2)}$ for $MoSe_2$ monolayers

To experimentally estimate $\chi^{(2)}$ of PVD-grown $MoSe_2$ monolayers, we used a 0.5 mm thick z-cut quartz crystal as our reference sample. SHG measurements on both quartz and $MoSe_2$ monolayers were carried out consecutively on the same day, using the same optical alignment and laser pulse conditions, to eliminate the impact of any unintentional difference in measurement conditions. See Figure S4.

Considering the layered structure of $MoSe_2$ monolayers and a bulk single-crystalline quartz, the ratio of second order nonlinear susceptibilities of a $MoSe_2$ monolayer to that of quartz is given by [4,5]:

$$\frac{\chi_{MoSe_2}^{(2)Sheet}}{\chi_q^{(2)}} = \frac{L_q \,\mathrm{sinc}\left(\frac{\Delta k_q \;L_q}{2}\right)}{|\beta(\omega)|\, n_q(\omega)\sqrt{n_q(2\omega)}}\sqrt{\frac{I_{MoSe_2}(2\omega)}{I_q(2\omega)}}$$

Here, $\chi_q^{(2)} = 0.6$ pm/V [5], L_q is the effective interaction length, Δk_q is the frequency dependent phase mismatch for quartz, $\beta(\omega)$ is the structure factor for $MoSe_2$ monolayers on SiO_2/Si, and $n_q(\omega)$ and $n_q(2\omega)$ are the refractive indices of quartz at the fundamental (λ= 800 nm) and SH (λ/2= 400 nm) frequencies, respectively [6]. We took L_q to be identical to the coherence buildup length [5,7,8], which is calculated to be L_q= 0.14 μm for quartz. Using these values, we estimated the second order sheet non-linear susceptibility of $MoSe_2$ monolayers, $\chi_{MoSe_2}^{(2)Sheet}$ to be about 1.7 ×

10^6 pm^2/V. Using $\chi^{(2)}_{MoSe_2} = \frac{\chi^{(2)Sheet}_{MoSe_2}}{\Delta h}$ and Δh = 0.645 nm (thickness of $MoSe_2$ monolayers), we determined the second order nonlinear susceptibility of $MoSe_2$ to be approximately 2,700 pm/V.

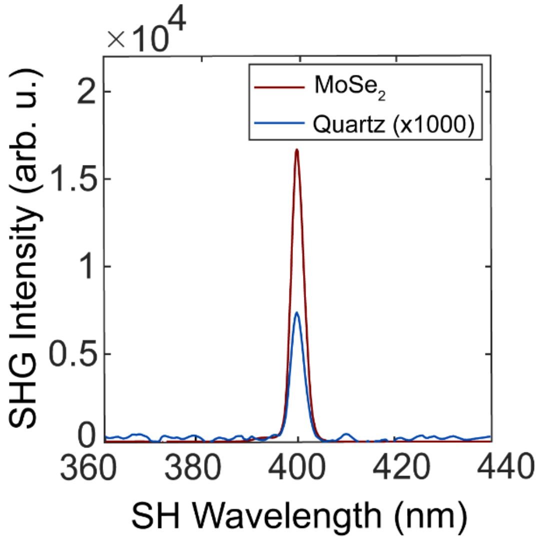

Figure S4. Comparison of SHG Intensity of $MoSe_2$ with quartz. Comparative SHG intensity plot of PVD-grown $MoSe_2$ monolayers and quartz using an 800 nm pump laser.

5. Optical images of $MoSe_2$ monolayers on SiO_2/Si with different thicknesses

$MoSe_2$ monolayers were grown on SiO_2 with the following thicknesses: 50 nm, 87 nm, 200 nm, 300 nm under identical growth conditions. The color of the islands seen in Figure S5 is different due to the change in interference coming from the different thicknesses of the SiO_2 substrate.

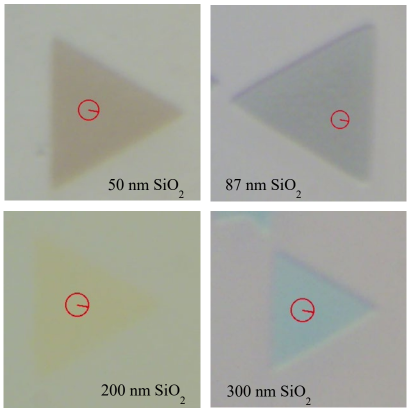

Figure S5. Optical images of $MoSe_2$ monolayers grown on SiO_2 of different thicknesses.

6. Thickness dependence (SiO_2 substrate) on the second harmonic generation

SHG measurements were carried on the $MoSe_2$ monolayers grown on substrates with four different thicknesses: 50 nm, 87 nm, 200 nm, and 300 nm SiO_2. We used an 800 nm frequency on the pump laser. The SHG counts measured from $MoSe_2$ monolayers as a function of SiO_2 thickness, shown as an inset in Fig 4(d) of the main manuscript, match the trend observed in the interference simulation. As shown in Table T1, a relative SHG enhancement attributed to biaxial tensile strain consistently exceeds a factor of 2. The four SHG measurements were performed consecutively on the same day, using the same optical alignment and laser pulse conditions, to eliminate the impact of any unintentional difference in measurement conditions.

Table T1. Estimation of SHG enhancement from biaxial strain. The SHG enhancement (Strain + Interference) is the overall SHG enhancement factor determined experimentally, the SHG enhancement (Interference) is obtained from the simulation, and the SHG enhancement (Strain) is extracted by excluding the enhancement factor from interference from the total SHG enhancement. Here, we use SHG intensity from $MoSe_2$ monolayers on a 73 nm Si_3N_4/Si substrate as a normalizing factor.

Thickness of SiO_2 (nm)	50	87	200	300
SHG enhancement (Strain + Interference) = $\frac{I_{SHG}(\text{d nm } SiO_2)}{I_{SHG}(\text{73 nm } Si_3N_4)}$	9.65	17.95	12.04	0.77
SHG enhancement (Interference)	1.96	7.21	4.74	0.21
SHG enhancement (Strain)	4.9	2.49	2.54	3.67

References

1 Haley, Kristine L., et al. "Heated assembly and transfer of van der Waals heterostructures with common nail polish." *Nanomanufacturing* **2021**, 1, 49-56.

2 Island, Joshua O., et al. "Precise and reversible band gap tuning in single-layer $MoSe_2$ by uniaxial strain." *Nanoscale* **2016**, 8, 2589-2593.

3 Tonndorf, Philipp, et al. "Photoluminescence emission and Raman response of monolayer MoS_2, $MoSe_2$, and WSe_2." *Optics Express* **2013**, 21, 4908-4916.

4 Song, Ying, et al. "Interference tunable second harmonic generation for two-dimensional materials in layered structures." *Optics Express* **2023**, 31, 19746-19753.

5 R. Boyd, Nonlinear Optics, Fourth Edition (Academic Press, 2020).

6 Malitson, I. H., "Interspecimen Comparison of the Refractive Index of Fused Silica", J. Opt. Soc. Am. **1965**, 10, 1205.

7 Mlejnek, M., Wright, E. M., Moloney, J. V., & Bloembergen, N., "Second Harmonic Generation of Femtosecond Pulses at the Boundary of a Nonlinear Dielectric", Phys. Rev. Lett. **1999**, 83, 2934.

8 Mankowsky, R., von Hoegen, A., Först, M., & Cavalleri, A. "Ultrafast reversal of the ferroelectric polarization". Phys. Rev. Lett., **2017**, 118, 197601.
